# Curcumin Analog DM-1 in Monotherapy or Combinatory Treatment with Dacarbazine as a Strategy to Inhibit *In Vivo* Melanoma Progression

**DOI:** 10.1371/journal.pone.0118702

**Published:** 2015-03-05

**Authors:** Fernanda Faião-Flores, José Agustín Quincoces Suarez, Andréa Costa Fruet, Silvya Stuchi Maria-Engler, Paulo Celso Pardi, Durvanei Augusto Maria

**Affiliations:** 1 Department of Clinical Chemistry & Toxicology, School of Pharmaceutical Sciences, University of São Paulo, São Paulo, Brazil; 2 Laboratory of Organic Synthesis, Anhanguera University of São Paulo, UNIAN, São Paulo, Brazil; 3 Laboratory of Experimental Pathology, Anhanguera University of São Paulo, UNIAN, São Paulo, Brazil; 4 Laboratory of Biochemistry and Biophysics, Butantan Institute, São Paulo, Brazil; IDI, Istituto Dermopatico dell’Immacolata, ITALY

## Abstract

Malignant melanoma is a highly aggressive form of skin cancer with a high mortality rate if not discovered in early stages. Although a limited number of treatment options for melanoma currently exist, patients with a more aggressive form of this cancer frequently decline treatment. DM-1 is a sodium phenolate and curcumin analog with proven anticancer, anti-proliferative and anti-metastatic properties. In this paper, the DM-1 compound showed in vivo antitumor activity alone or in combination with chemotherapeutic DTIC in B16F10 melanoma-bearing mice. Beneficial effects such as melanoma tumor burden reduction with pyknotic nuclei, decreased nuclei/cytoplasmic ratio and nuclear degradation occurred after DM-1 treatment. No toxicological changes were observed in the liver, kidneys, spleen and lungs after DM-1 monotherapy or DTIC combined therapy. DTIC+DM-1 treatment induced the recovery of anemia arising from melanoma and immunomodulation. Both DM-1 treatment alone and in combination with DTIC induced apoptosis with the cleavage of caspase-3, -8 and -9. Furthermore, melanoma tumors treated with DM-1 showed a preferential apoptotic intrinsic pathway by decreasing Bcl-2/Bax ratio. Considering the chemoresistance exhibited by melanoma towards conventional chemotherapy drugs, DM-1 compound in monotherapy or in combination therapy provides a promising improvement in melanoma treatment with a reduction of side effects.

## Introduction

Skin cancer is the most common human cancer worldwide and melanoma is a highly aggressive form of this cancer. Cancer-related deaths because of metastatic melanoma account for about 75% [1]. Although a limited number of treatment options for melanoma currently exist, patients with a more aggressive form of disease for which no effective/long-lasting treatment presently exists frequently decline treatment [2].

Before 2011, dacarbazine (DTIC), recombinant human interleukin-2 and high dose interferon-alpha were the treatments approved by the FDA for metastatic melanoma. Together, these treatments generated tumor responses only in around 20% of patients, often with no benefit on progression free survival or overall survival [3].

According to National Comprehensive Cancer Network (NCCN) guidelines, metastatic melanoma, irrespective of genotype status (BRAF or KIT mutations), can be treated with DTIC, temozolomide and paclitaxel, however, these treatments are not target-specific like BRAF and MEK inhibitors [4].

Malignant melanoma cells exhibit enhanced survival and proliferation abilities. One of the most important reasons for this is their anti-apoptosis capacity, which is the predominant problem for clinical chemotherapy drug tolerance [5].

Curcumin presents preclinical and clinical studies as an antitumor agent [6]. The low potency, multiple biological effects and low bioavailability of curcumin have led to the development of curcumin analogs with similar safety profiles but increased anticancer activity and solubility [2].

DM-1 is a curcumin analog which has been previously studied in animal models of melanoma and breast adenocarcinoma [7,8]. This compound is a powerful anticancer agent with anti-proliferative and anti-metastatic activities, with minimal side effects on normal tissue due to its high selectivity for tumor cells without any significant cytotoxic effect on normal cells [9,10].

In the present study, we analyzed the action mechanism after DM-1 treatment in monotherapy or in combination with dacarbazine (DTIC) in B16F10 melanoma-bearing mice by means of its inhibitory tumor effects and apoptotic cell death with minimal side effects.

## Materials and Methods

### Cell lines and culture conditions

The B16F10 murine melanoma cell line was purchased from the American Type Culture Collection (Manassas, USA). The cells were grown in 75 cm^2^ flasks with DMEM medium (Cultilab, Brazil) supplemented with 10% heat-inactivated fetal bovine serum (Cultilab, Brazil), 2 mM L-glutamine (Sigma Chemical Company, USA) and 0.1 g/mL streptomycin (FontouraWyeth AS, USA) at 37°C in a 5% CO_2_ atmosphere.

### DM-1: Sodium 4-[5-(4-hydroxy-3-methoxyphenyl)-3- oxo-penta-1,4-dienyl]-2-methoxy-phenolate

**Fig 1:**
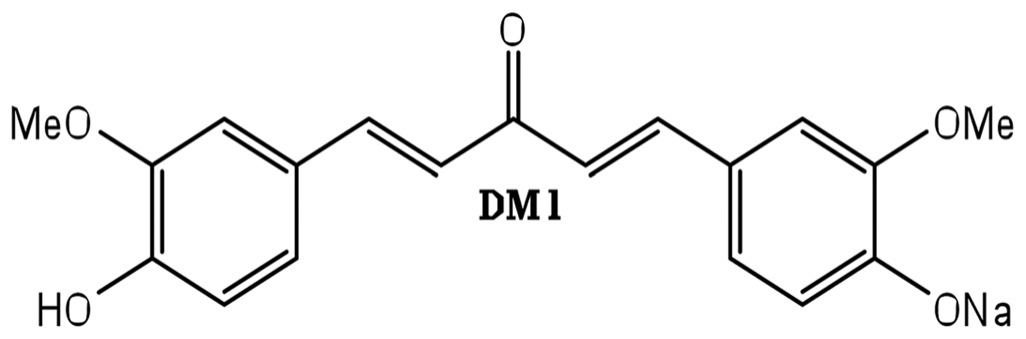
Molecular structure of DM-1.

Dried sodium ethanolate (0.01 mol) was mixed with 1,5-bis(4-hydroxy-3-methoxyphenyl)-1,4-pentadien-3-one [11] (0.01 mol; 3.26 g) in a 1:1 molar ratio and stirred at room temperature under anhydrous reaction conditions, followed by solvent rotoevaporation until solidification. The compound C_19_H_17_O_5_Na has a molecular weight of 348g (Fig. 1). Results of the structural characterization of the isolated compound were the same as those described previously by our group [12,13].

### Ethic Statement

Male C57BL/6J mice (weighing 28–30 g) from the Butantan Institute, São Paulo, SP, Brazil, were housed in standard environmental conditions (21°C, 60%-70% humidity) under a 12-hour light/dark cycle and air filtration. The animals had free access to water and food.

All mice were sacrificed by cervical dislocation at the 14th day of treatment or when they presented any unusual behavior, such as restricted body mobility or difficulty to eat or drink.

Mice were monitored three times a week for welfare condition and potential discomfort, therefore, no analgesics or anesthetics were applied. This study was carried out in strict accordance with the recommendations in the Guide for the Care and Use of Laboratory Animals of the National Institutes of Health. The protocol was approved by the Ethical Committee for Animal Research at the Butantan Institute (Permit Number: 590/09).

### Inoculation of B16F10 melanoma cells in mice

B16F10 melanoma cells were trypsinized and viable cells were counted by trypan blue dye exclusion. For tumor inoculation, 5×10^4^ cells were suspended in 100 μl of phosphate buffered saline (PBS) and injected subcutaneously into the left flank regions of C57BL/6J mice.

### *In vivo* antitumor evaluation

B16F10 melanoma-bearing mice were randomly assigned into four groups of 5 animals each on the fourteenth day after tumor inoculation. The DM-1 compound was administered daily (83 μM) considering the IC50 value determined in the in vitro assay [8] and DTIC was administered every two days (total of seven doses) of 4.5 mg/kg/body per mice [14]. Both treatment were administered intraperitoneally. Besides, one group of C57BL/6J mice that did not receive B16F10 melanoma cells inoculation (Normal group) was performed to compare normal parameters. The B16F10 melanoma-bearing mice groups were assigned in:

**Control:** B16F10 melanoma-bearing mice treated daily with 100 μL of saline solution;
**DTIC:** B16F10 melanoma-bearing mice treated only with DTIC diluted in saline solution;
**DM-1:** B16F10 melanoma-bearing mice treated daily only with DM-1 compound diluted in saline solution;
**DTIC+DM-1:** B16F10 melanoma-bearing mice treated with combination of DM-1 compound (daily) plus DTIC (every two days for seven doses).

Blood samples were collected from mice retro-orbital sinuses with heparinized capillary tubes. Red blood cells (RBC), white blood cells (WBC), reticulocytes and platelets were manually counted using a Neubauer hemocytometer chamber via phase microscopy [15]. Side effects included increase of several tissue-damage-related enzyme activities in plasma, including aspartate aminotransferase (AST), alanine aminotransferase (ALT), urea and creatinine were analyzed by LABORLAB kinetic assay (00200-ALT; 00300-AST; 02800-Urea and 01600-Creatinine).

### Macroscopic assessment of melanoma

Tumor sizes were measured daily using a caliper-like instrument. The longest length and the length perpendicular to the longest length were multiplied to obtain the tumor size (area) in cm^2^ [16]. Necropsies were performed 28 days after tumor inoculation.

### Microscopic assessment of melanoma

For histopathologic evaluation, representative transversal samples of the tumors were collected, embedded in paraffin, stained with hematoxylin and eosin, and viewed via light microscopy (magnification ×40).

### Western blotting

Small fragments of different areas of the tumor from each group were washed and total cell lysates were obtained with Laemmli Buffer (10% SDS, 0.0625 M Tris-HCl pH 6.8, 10% glycerol, and 5% 2-beta-mercaptoethanol) with protease inhibitor cocktail (Roche 11836170001) extraction. After that, 40 μg of total protein were subjected to electrophoresis in 15% gradient SDS gels under reducing conditions, and subsequently transferred to polyvinylidenedifluoride (PVDF) membranes (Hybond-P, Amersham Pharmacia Biotech, Piscataway, NJ, USA). The membranes were blocked in 5% dry milk diluted in TBS-T (50 mM Tris-HCl pH 7.5, 150 mMNaCl, 0.1% Tween-20) for 1 h and then incubated overnight with the following antibodies: α-tubulin, Bax, Bcl-2, caspase-3, caspase-8 and caspase-9 (Cell Signaling, USA). Protein bands were detected by an enhanced chemiluminescence system ECL (Amersham Pharmacia Biotech). Information about the western blotting antibodies used can be found in the supplementary data (S1 Table). The α-tubulin antibody was used as a loading control.

### Statistical analysis

Results are expressed as mean ± s.d. Data were analyzed using one-way analysis of variance (ANOVA) and significant mean differences were determined using multiple comparisons by the TUKEY-KRAMER test at the p<0.05 level. Significant differences between the control and treated groups are indicated by ***p<0.001, **p<0.01 and *p<0.05.

## Results

### Survival rate and tumor mass growth

The DM-1 compound showed *in vivo* antitumor activity. The cytotoxic effects were obtained with DM-1 compound administered alone or in combination with chemotherapeutic DTIC. The B16F10 melanoma-bearing mice without treatment (control group) showed a significant tumor area increase. The group receiving DTIC chemotherapy alone showed a 43% tumor burden decrease in comparison to the control group. The group treated with DM-1 compound alone and the group treated with both therapies showed 57% and 63% tumor burden reduction, respectively, compared to the control group. Both groups treated with DM-1 compound showed better antitumor effects than DTIC monotherapy. Furthermore, twenty-eight days after B16F10 melanoma cell inoculation, there were no live animals in the control group. The group treated with DTIC only presented 40% survival. The survival rate of both DM-1 monotherapy or in combination with DTIC was significantly increased in comparison to the control group or DTIC monotherapy, with 100% survival. These preliminary results were published in Faião-Flores (2013a) [9] and all complementary results are presented here.

### Macroscopic aspects, tumor mass and body weight evaluation after DM-1 monotherapy or DTIC combinatory treatment

**Fig 2:**
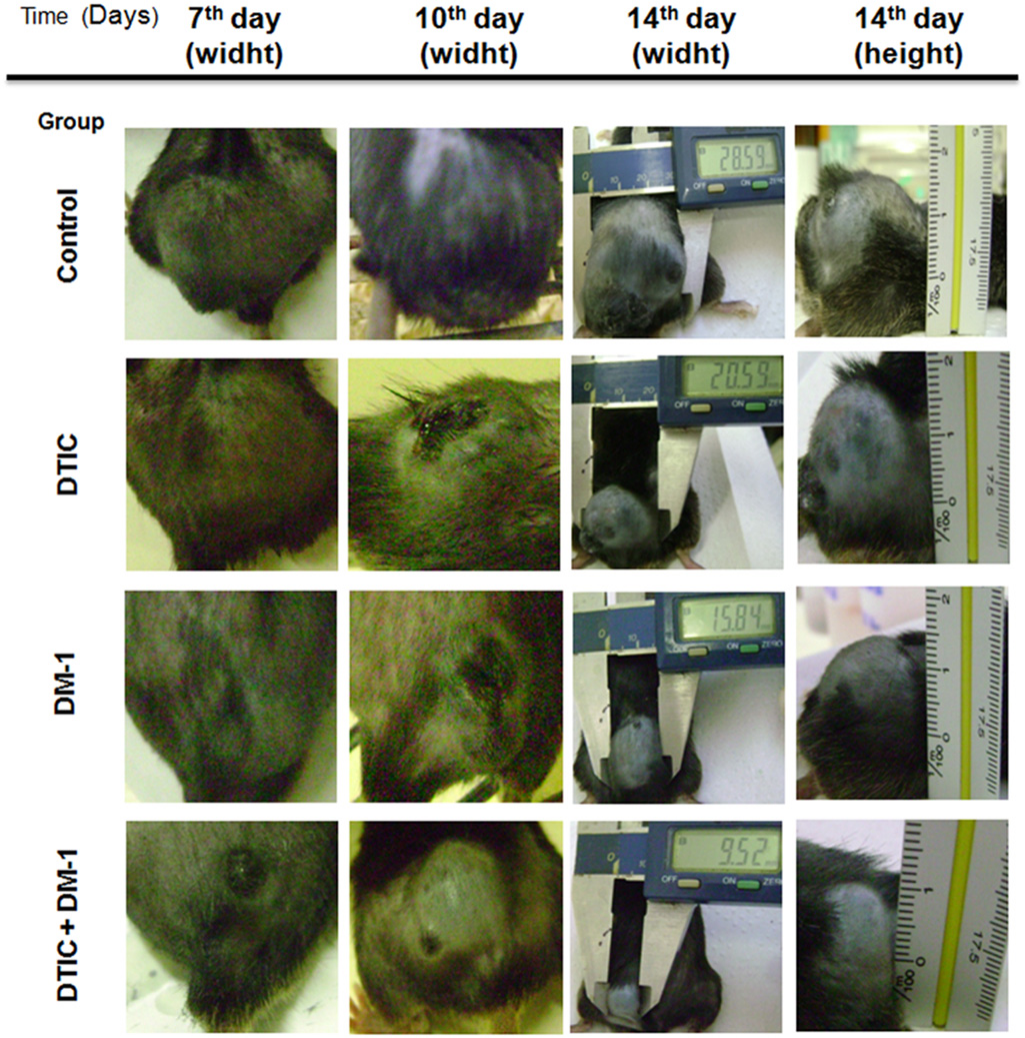
Macroscopic aspect of B16F10 melanoma-bearing mice on the 7^th^, 10^th^ and 14^th^ days of treatment with DTIC, DM-1 or DTIC+DM-1 in comparison to control group. The measurements were made using a caliper rule.

During the fourteen days of treatment, the B16F10 melanoma-bearing mice were photographed for macroscopic analysis of their dorsal tumors. DTIC and DM-1 groups presented smaller tumor sizes than the control group (about 28% and 45% reduction, respectively) while the combination therapy showed an inhibitory effect of 67% of tumor area/burden on the 15^th^ day after starting treatment (Fig. 2).

**Fig 3:**
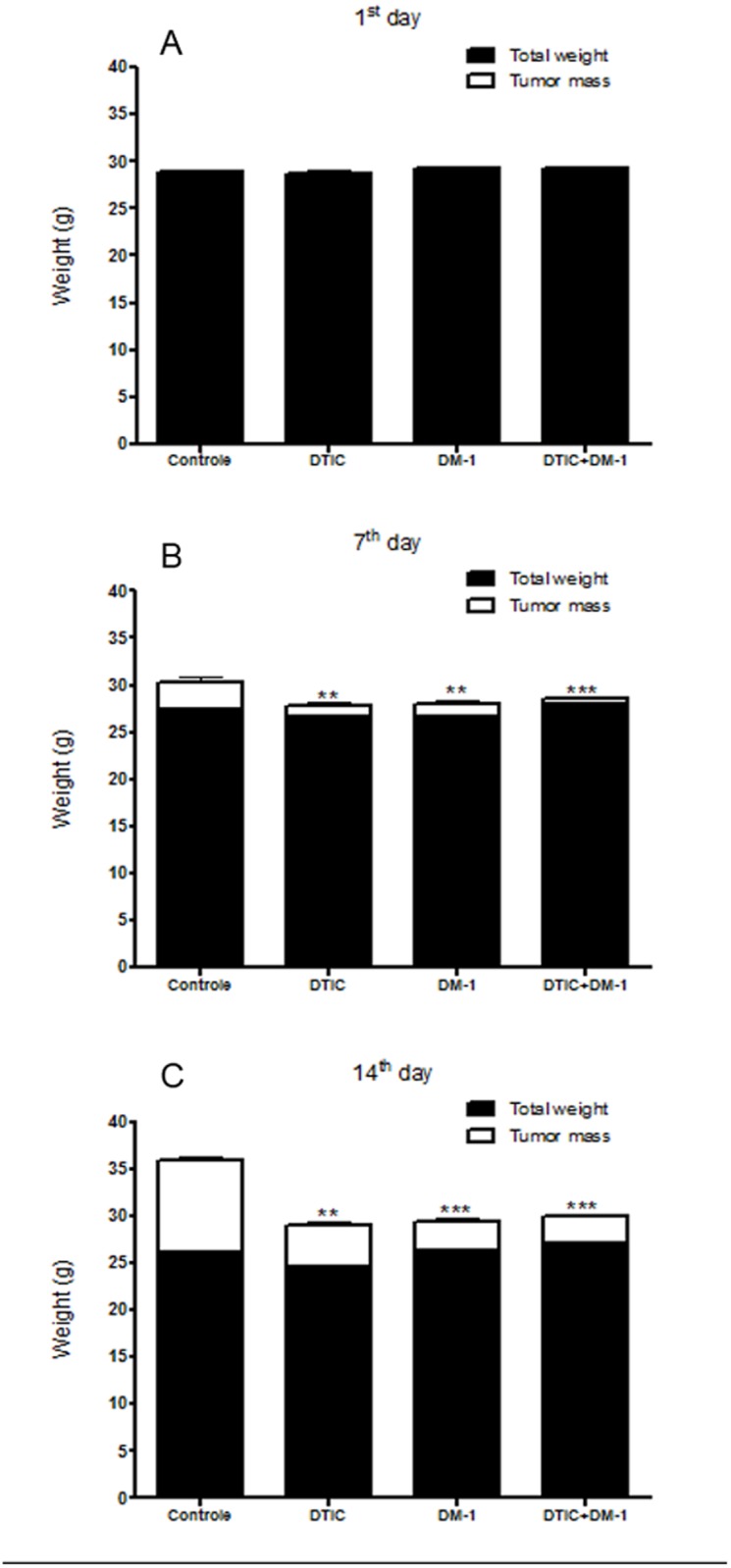
Total weight and tumor mass of B16F10 melanoma-bearing mice on the 1st day of DTIC, DM-1 or DTIC+DM-1 treatment in comparison to the control group (A); Total weight and tumor mass of B16F10 melanoma-bearing mice on the 7^th^ day of DTIC, DM-1 or DTIC+DM-1 treatment in comparison to the control group(B); Total weight and tumor mass of B16F10 melanoma-bearing mice on the 14^th^ day of DTIC, DM-1 or DTIC+DM-1 treatment in comparison to the control group (C). The values are expressed as mean ± s.d. Significance is indicated by *p<0.05, **p<0.01 and ***p<0.001 compared to control.

The B16F10 melanoma-bearing mice showed the same profile on the first day in the control or treatment groups (Fig. 3a). However, on the 7^th^ day of the experiment, the control group presented increased values of tumor mass compared to the DM-1, DTIC or DTIC+DM-1 groups, which showed no significant loss or increase in total weight (Fig. 3b). After the 14^th^ day, control group mice showed a tumor mass of about 28% of the total body weight, while the DTIC group showed 17% for the same parameter. However, treatment with DM-1 in monotherapy or combination therapy showed tumor mass <10% of total body weight and the absence of tumor ulcerations (Fig. 3c).

### Melanoma morphology alteration mediated by DM-1

**Fig 4:**
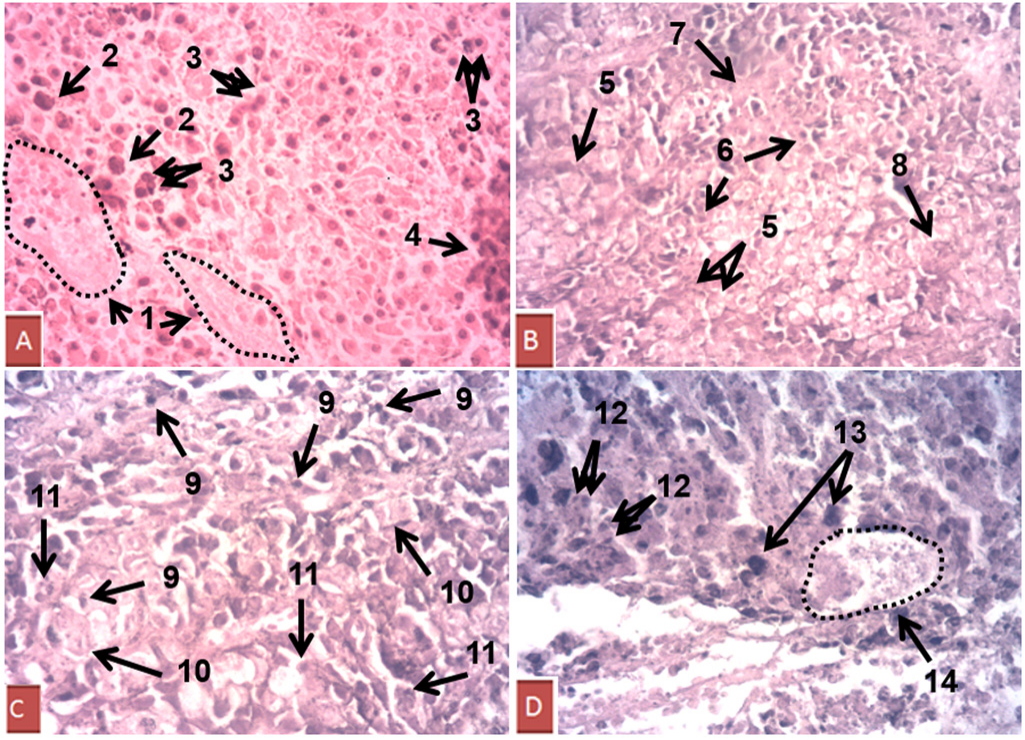
Hematoxylin/eosin-stained tumor sections from B16F10 melanoma-bearing mice. The control group (A) showed singular characteristics such as 1) large blood vessels, 2) nuclear hyperchromasia, 3) multinucleation and 4) proliferating cell cluster. The DM-1 group (B) presented 5) loss of cytoplasmic limits, 6) pyknotic nuclei, 7) decreased nuclei/cytoplasmic ratio and 8) nuclear degradation. The group treated with DTIC+DM-1 (C) also showed 9) pyknotic nuclei, 10) loss of cytoplasmic limits and 11) nuclear hypochromasia. The DTIC group (D) showed 12) multinucleation, 13) karyomegaly and 14) large blood vessels. Magnification: ×40.

Melanoma tissues were analyzed to verify possible morphological changes in cell density, nuclei alteration and cell death signals. The control group showed singular characteristics such as large blood vessels, nuclear hyperchromasia, multinucleation and proliferating cell clusters, which are associated with intense proliferative activity and conventional criteria for malignancy. The DTIC group also showed multinucleation and large blood vessels, as well as karyomegaly, all of which are correlated to increased proliferation. The DM-1 group presented a loss of cytoplasmic limits, pyknotic nuclei, decreased nuclei/cytoplasmic ratio and nuclear degradation, which are clear signals of cell death, mainly by apoptosis. The group treated with DTIC+DM-1 also showed a significant quantity of pyknotic nuclei, with a loss of cytoplasmic limits, evident nuclear hypochromasia and debris formation (Fig. 4).

### Immunomodulation after DM-1 treatment

**Fig 5:**
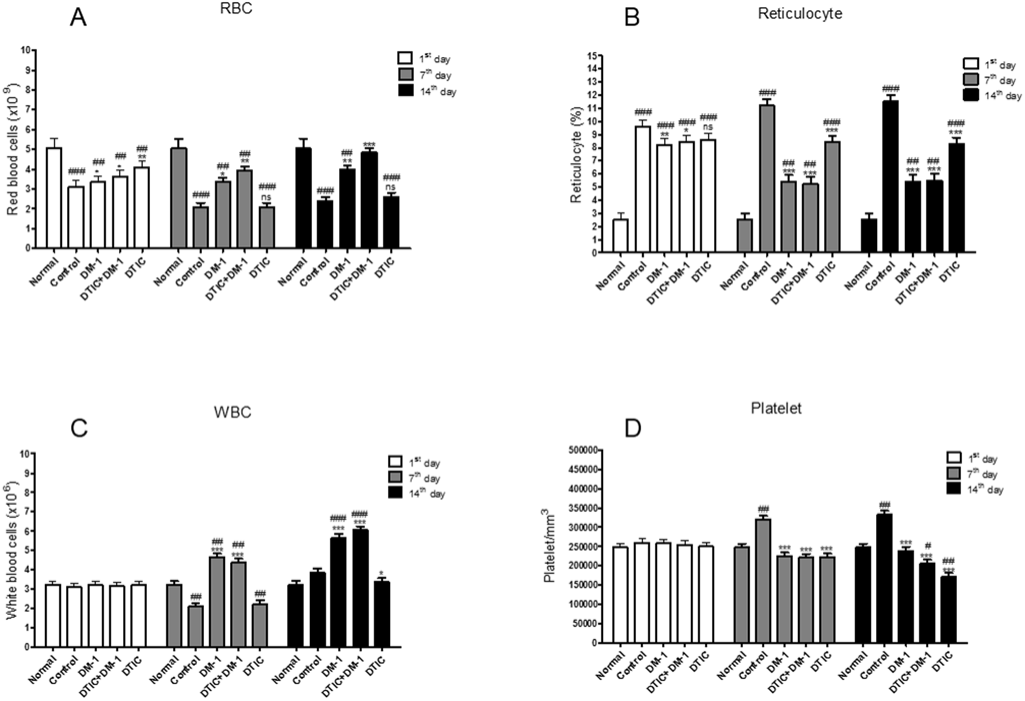
Red blood cells (RBC) (A); reticulocytes (B); white blood cells (WBC) (C) and platelets (D) in peripheral blood of untreated normal mice and B16F10 melanoma-bearing mice after DTIC, DM-1 or DTIC+DM-1 treatment. The peripheral blood was collected and the cells were counted on the 1st, 7th and 14th days of treatment. The values are expressed as mean ± s.d. Significance is indicated by: *p<0.05, **p<0.01 and ***p<0.001 compared to control; and #p<0.05, ##p<0.01 and ###p<0.001 compared to untreated normal mice.

After 14 days of treatment, B16F10 melanoma-bearing mice of the control group presented a severe reduction in the total number of red blood cells (RBC) (approximately 45% compared to non-tumor-bearing mice or the normal group). The mice treated with DTIC+DM-1 showed similar values to the normal group, with a recovery of anemia arising from melanoma (Fig. 5a).

At the beginning of the treatment protocols, all B16F10 melanoma-bearing mice had up to 4x more reticulocytes in the peripheral blood than normal mice. In the middle of the treatment (7^th^ day), the DM-1-treated groups showed a significant reduction of this parameter, while the control group still maintained a high quantity. At the end of the treatment (14^th^ day), the DTIC-treated mice still showed approximately 3.5x more reticulocytes than the normal group, with levels approximately 33% higher than for mono- or combination therapy with DM-1 (Fig. 5b).

DM-1-treated mice showed a significant increase in the total number of white blood cells (WBC) after the 7^th^ day of treatment, while the DTIC and control groups showed the same decrease in this parameter in the same period. After this time, the increased WBC profile was maintained in the DM-1 group, indicating immunomodulation (Fig. 5c).

The control group exhibited a significant increase in platelet number. On the other hand, the groups treated with chemotherapeutic DTIC, DM-1 compound or combination therapy were responsible for a decline of this parameter, comparing the DM-1-treated mice platelet levels to those in normal mice (non-tumor bearing animals) (Fig. 5d).

### Toxicity reduction after DM-1 treatment

**Fig 6:**
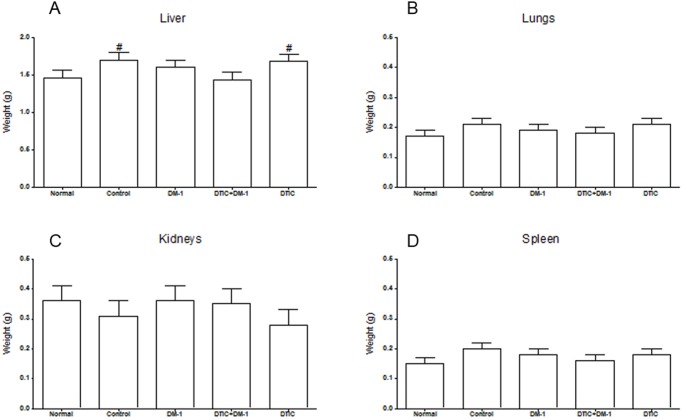
Liver (A); lungs (B); kidneys (C) and spleen (D) weight of untreated normal mice and B16F10 melanoma-bearing mice after DTIC, DM-1 or DTIC+DM-1 treatment. The values are expressed as mean ± s.d. Significance is indicated by #p<0.05 compared to untreated normal mice.

The main internal organs (liver, lungs, kidneys and spleen) of B16F10 melanoma-bearing mice were evaluated macroscopically and by weight. Control and DTIC groups presented moderate hepatomegaly after the endpoint. The DM-1 compound acted as a cytoprotective agent in the combination therapy with DTIC, because the liver retained similar morphological conditions to normal mice (non-tumor-bearing animals). All other organs (lungs, kidneys and spleen) showed no significant changes in their weight and macroscopic morphology (Fig. 6). Furthermore, biochemical parameters analysis such as hepatic biomarkers alanine and aspartate transferases, kidney biomarkers urea and creatinine showed similarity in DM-1 groups compared to normal mice, whereas DTIC group presented elevated levels of these parameters, indicating side effects corroborating with hepatomegaly. Furthermore, there was an increase of nitrogenous end products of metabolism (urea and creatinine) that could affect liver function (S1 Fig.).

### Targeting apoptosis pathway as a strategy for melanoma therapy

**Fig 7:**
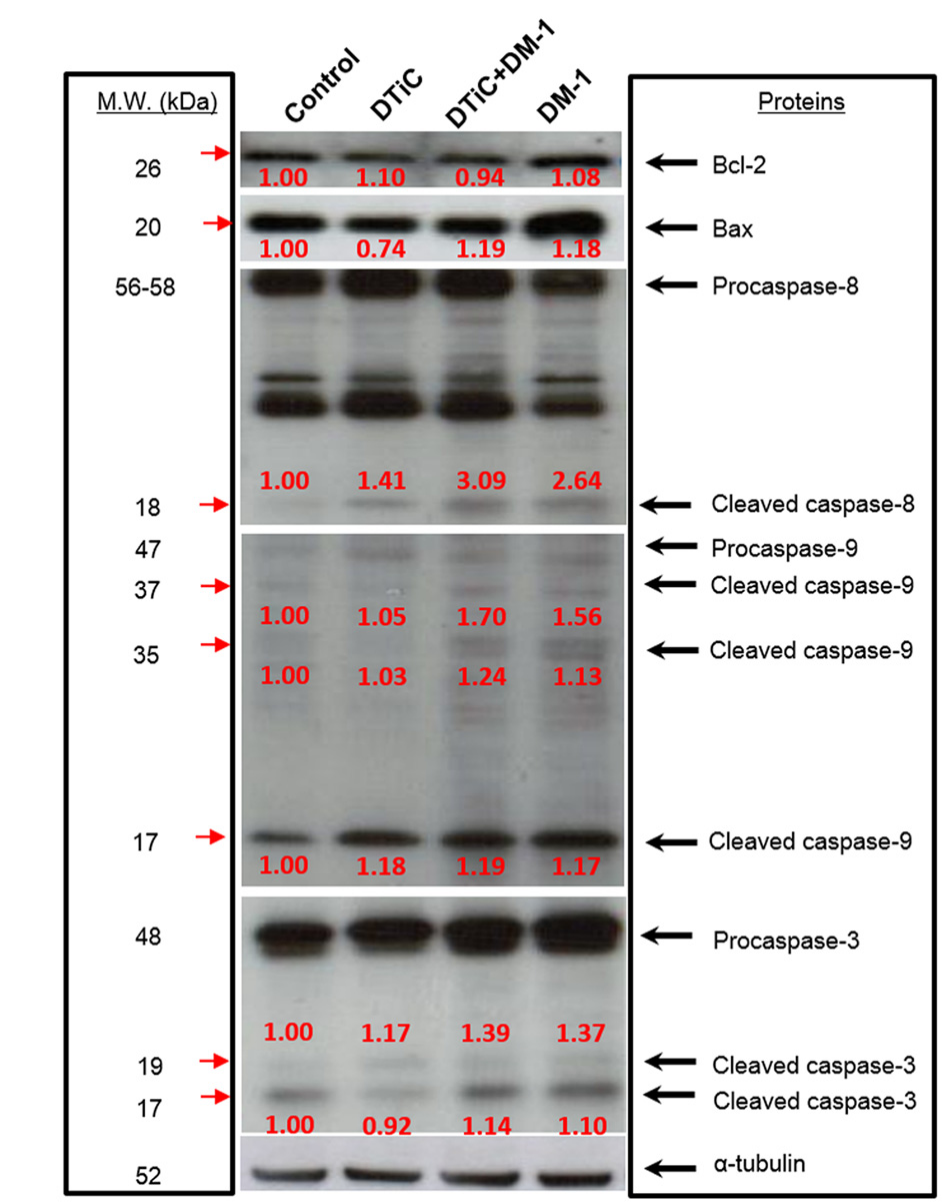
Involvement of apoptotic members in melanoma tissues samples of B16F10 melanoma-bearing mice. Protein total extracts were used to analyzed Bcl-2, Bax, caspase-8, caspase-9 and caspase-3 after DTIC, DTIC+DM-1 or DM-1 treatment at the endpoint of the experiment. α-Tubulin expression was used as a loading control.

All treated groups presented an increase in caspase-8 cleavage when compared to controls, mainly in the DM-1 treatments. In the DM-1 groups, the Bcl-2/Bax ratio was decreased, with a significant increase in the pro-apoptotic Bax protein level (S2 Fig.). Furthermore, the intrinsic apoptosis pathway was also confirmed by caspase-9 cleavage in all treatments (17 kDa fragment) and mainly in DM-1 groups (35 kDa and 37 kDa fragments), with higher expression in DTIC+DM-1 group. As evidence of this, there was also caspase-3 cleavage (19 kDa and 17 kDa fragments) with higher expression in the DM-1 mono- and combination therapy groups (Fig. 7).

## Discussion

Malignant melanoma, the major fatal form of skin cancer, is an aggressive and refractory cancer derived from melanocytes [17]. Currently, the incidence of melanoma has been continuously increasing worldwide and has become a huge public health issue. Surgery, radiation and chemotherapy are the standard means of treating melanoma [18], but its strong resistance regulated by several heterogeneous factors and high mortality rates are the correlated characteristics of this cancer [5]. Due to this complexity, tumor cells are susceptible to different types of targeted therapy, but, unfortunately, single agents often have limited efficacy due to intrinsic or acquired resistance mechanisms [19].

Since the discovery of activating mutations in the BRAF oncogene and the stimulation of an immune-mediated antitumor response in melanoma, there has been remarkable progress in the development of targeted therapies for unresectable and metastatic melanoma [20]. Although vemurafenib, dabrafenib (BRAF mutation inhibitors) and trametinib (MEK inhibitor) have been associated with impressive clinical results, the majority of patients relapsed quite rapidly with a median duration of response of 6 months [19]. Furthermore, a significant percentage of patients showed intrinsic resistance e.g. acquired resistance by extracellular signal-regulated kinases (ERK) reactivation, MEK mutations or PI3K/Akt activation [21,22].

BRAF inhibition is also associated with complications of cutaneous squamous cell carcinomas, which is an on-target adverse effect resulting from the paradoxical activation of the MAPK pathway in cells with wild type BRAF and strong upstream signaling in the MAPK pathway and is of particular relevance when combined with immunotherapies [23]. It is becoming evident that the effects of paradoxical MAPK pathway activation by BRAF inhibitors in non-BRAF-mutant cells needs to be taken into account, which may be implicated in the problems encountered in the first clinical trial testing a combination of the BRAF inhibitor vemurafenib with ipilimumab (anti-CTLA4), which revealed significant liver toxicity [24].

DM-1 is a powerful antitumor agent with proven anti-metastatic and anti-proliferative activities. Its pharmacological activity is selective for tumor cells (*in vitro* results) and restricted for tumor tissue (*in vivo* results), with minimal side effects on the normal and surrounding tissues [8,9]. DM-1 compound also showed *in vivo* antitumor activity after administration alone or in combination with chemotherapeutic DTIC in B16F10 melanoma-bearing mice [9]. Both groups, DM-1 monotherapy and DTIC+DM-1, revealed a tumor burden reduction of about 60%, partly via cell cycle arrest due to cyclin D1 and Ki67 decrease [8]. Besides, this group showed a significant increase of survival rate in comparison to the control or DTIC monotherapy groups. Additionally, the tumor mass was also reduced in the DM-1 treated groups, showing a tumor mass less than 10% of total body weight and without ulcerations, both of which are associated with intratumoral blood vessel density due to a rich vascular supply that prompts tumor cell dissemination, and also indicate poor prognosis [25].

Samples of melanoma tissues were analyzed to verify possible morphological changes in cell density, nuclei coloration and cell death signals. The control and DTIC groups showed characteristics such as multinucleation, nuclear hyperchromasia, karyomegaly and proliferating cell cluster. Previous studies have demonstrated that abnormal mitotic figures, variations in the degree of chromasia and variations from cell to cell are signals of genetic instability and can demonstrate the high proliferation and aggressive profile reflecting tumor progression [26–27]. The DM-1 treatment in monotherapy or combined therapy with DTIC showed a loss of cytoplasmic limits, nuclear hypochromasia, pyknotic nuclei, decreased nuclei/cytoplasmic ratio and nuclear degradation. Burgoyne defined pyknosis as an intense nuclear condensation with intense refractivity and stated that is was related to apoptosis in vertebrate cells. Moreover, a decrease in the nuclear to cytoplasmic ratio is present in inactivated cells and revealed cell cycle arrest and cell death, mainly by apoptosis [28].

An evaluation of side effects is needed to proceed with direct interventions in patients receiving chemotherapy. Anemia has been identified as a predictive factor for decreased survival in settings other than oncology because it compromises therapy and hypoxic tumor cells exhibit resistance to chemotherapy. The availability of safe and effective therapies for anemia associated with cancer, coupled with recent evidence that anemia itself may well have an impact on survival, make it important that the potential of anemia therapy to improve survival outcomes in cancer patients be explored in clinical trials [29]. Additionally, ameliorating the anemia symptom may prolong patient survival due to enhancing tumor oxygenation, and thus increasing tumor sensitivity to chemotherapy or radiation [30]. At the end of treatment, control and DTIC groups showed RBC reduction compared to the normal group. In addition, DTIC-treated mice still showed more reticulocytes than normal mice, and levels around 33% higher than those treated with mono- or combination therapy with DM-1. The combinatory DTIC+DM-1 treatment showed a recovery of anemia induced by melanoma because they presented similar RBC values in comparison to the normal group. This event was attributed to the compound DM-1, because DM-1 presented RBC values close to baseline in monotherapy, whereas mice treated with chemotherapeutic DTIC exhibited similarities to the control group. Furthermore, RBC increase in the DM-1-treated groups was not associated with bone marrow exhausting work with the release of immature cells because this group presented an anemic recovery profile of melanoma-bearing mice.

Low cancer-response rate is also due to the development of multiple mechanisms for cancer cells to evade immune surveillance and suppress effector immune cell function in the tumor microenvironment [31]. Some evidence has suggested that targeted therapies can not only provide additive effects but can also sensitize the tumor cells to immune attacks. Improvement in the effector function of immune cells and metastatic melanoma has served as a prime example to illustrate the potential combinatorial benefits of combinatory therapies [24]. DM-1 induced WBC increase indicating immune response activation against the tumor cells, whereas the DTIC and control groups showed WBC decrease, favoring opportunistic infections and leading to a decrease in cancer-response rate.

A significant increase in the platelet number was reported, which is a phenomenon that is directly involved with metastasis via tumor cell arrest stabilization in the vasculature, tumor cell proliferation and extravasation, and enhancing tumor cell interaction with the extracellular matrix [32]. The melanoma control group exhibited increased platelet numbers, which was an effect that was counterbalanced by DM-1, DTIC or combined therapy treatment.

No changes were observed in the weight of lungs, kidneys and spleen, which are all important in the metabolism and excretion of xenobiotics [33], in all of the melanoma-bearing mice compared to the normal group, demonstrating that none of these treatments affects the weight of organs. Moreover, no changes were found on histopathological analysis of the same organs (data not shown), evidencing an absence of side effects. Systemic toxicity of a substance can also manifest itself by reducing the water and food consumption, behavioral changes and variations in organ weights [34]. Body weight is one of the parameters used in toxicological analysis to indicate the often early onset of toxic effects of a substance in animals. The absence of changes in physiological parameters emphasizes the low toxicity of the DM-1 compound and its combination with DTIC.

Chemotherapeutic agents commonly cause transient increases in the enzymes liver without any clinical evidence of liver impairment. However, when clinical toxicity occurs, the early diagnosis and withdrawal of the offending drug is imperative. The most important factors in early diagnosis are a high index of suspicion for drugs as a cause of liver injury and knowledge of the type of injury that a particular drug can cause [35]. It is known that DTIC may induce hepatotoxicity and here, in combination with DM-1, this abnormality was not found.

Resistance to apoptosis is one important hallmark of melanoma, and its reversal is a common goal across most preclinical combinatorial target therapy studies [36]. The extrinsic pathway of apoptosis is initiated by TNF-receptors, which activate caspase-8. The intrinsic pathway is initiated with an increase in the Bax/Bcl-2 ratio and the release of cytochrome c, with subsequent activation of caspase-9. The Bcl-2-family proteins are a group of molecules, related by structure and function, which play a key role in the regulation of intrinsic apoptosis. They include executioner proteins, such as the pro-apoptotic members (Bax and Bak) and anti-apoptotic members (Bcl-2, Bcl-XL and Mcl-1), which block the oligomerization of Bax and Bak, inhibiting their activity and protecting the cell from apoptosis [37]. Procaspase-3 is hydrolyzed and activated by caspase-8 or -9 and acts in DNA fragmentation of apoptosis [38]. In the present study, the expression of cleaved caspase-3, -8 and -9 was detected following DM-1 treatment and in combination with DTIC. Furthermore, melanoma tumors treated with DM-1 showed a decreased Bcl-2/Bax ratio. *In vitro*, DM-1 also induced apoptosis via the extrinsic pathway by TNF-R1 and cleaved caspase-8 increase and by the intrinsic pathway via Bcl-2/Bax ratio decrease, cytochrome-c release and the electric mitochondrial membrane potential decrease in melanoma cells [9,10].

The results presented here demonstrate how new agents such as DM-1 can trigger death receptors in combination with the inhibition of different pro-survival processes, inducing cell death, as a potentially effective antitumor strategy for melanoma treatment. Considering the chemoresistance exhibited by melanoma towards conventional chemotherapy drugs, DM-1 compound in monotherapy or in combination therapy provides promising improvements in the therapeutic approach to melanoma treatment.

### Conclusion

In the present study, we report that the therapeutic anticancer agent DM-1 presents tumor mass reduction, survival rate improvement and biological effects on the anemia symptoms related to cancer with a recovery of RBC and WBC levels and normalization of platelets and reticulocytes to normal benchmarks. The effects of DM-1+DTIC in combinatory therapy presented a better anti-tumor performance, with an increase in DM-1 anti-tumor effects, leading to apoptotic process in both intrinsic and extrinsic pathway. We concluded that DM-1 presents chemoprotective activity against melanoma, preferentially by *in vivo* apoptotic induction with caspase cleavage in tumor cells with better results in the combinatory therapy with DTIC, permitting an enhanced performance of both antitumor agents.

## Supporting Information

S1 Fig A: Alanine transaminase (ALT); B: Aspartate transaminase (AST); C: Urea and (D) creatinine in plasma blood of untreated normal mice and B16F10 melanoma-bearing mice after DTIC, DM-1 or DTIC+DM-1 treatment. Peripheral blood was collected and the enzymes were analyzed in the 1^st^, 7^th^ and 14^th^ days of treatment. The values are expressed as mean ± s.d. Significance is indicated by: *p<0.05, **p<0.01 and ***p<0.001 compared to control; and #p<0.05, ##p<0.01 and ###p<0.001 compared to untreated normal mice.
(TIF)

S2 Fig Bcl-2/Bax ratio expression in tumor samples. DTIC, DTIC+DM-1 and DM-1 tumor samples were compared to control group. The values are expressed as mean ± s.d. Significance is indicated by: *p<0.05 and **p<0.01 compared to control group.
(TIF)

S1 Table Antibodies used in western blotting experiments. (DOC)
