# Supplementary figures and images for: Curcumin Analog DM-1 in Monotherapy or Combinatory Treatment with Dacarbazine as a Strategy to Inhibit In Vivo Melanoma Progression
Source: PLoS One. 2015 Mar 5;10(3):e0118702. doi: 10.1371/journal.pone.0118702 (PMC4350837; doi:10.1371/journal.pone.0118702)

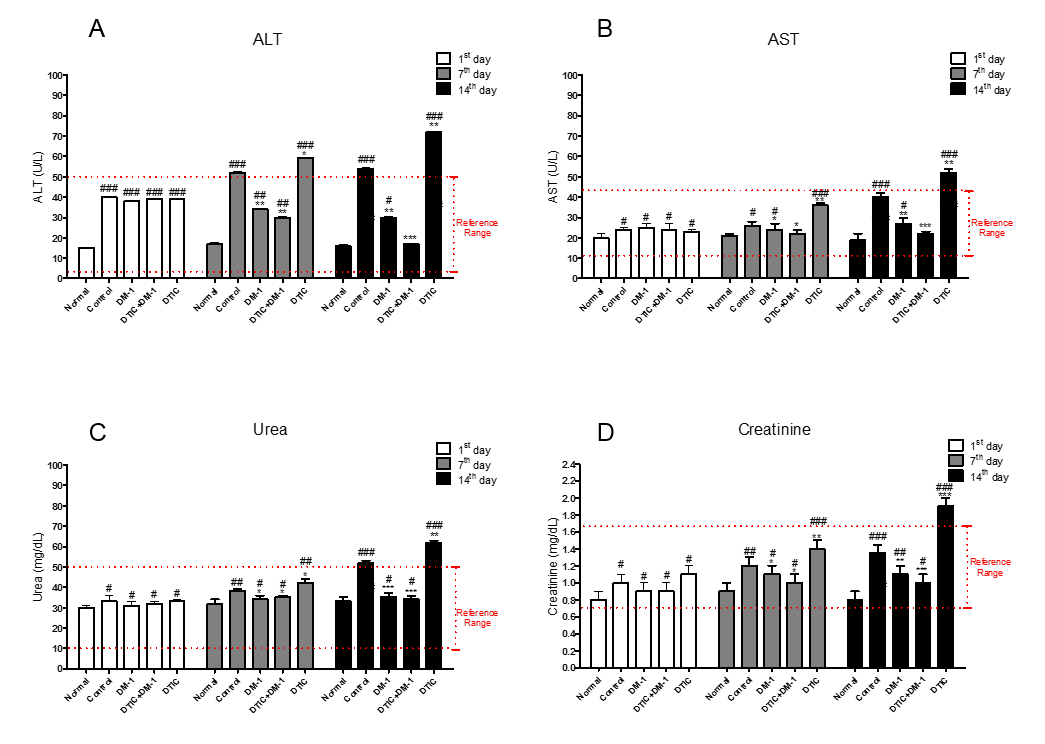

Supplement: S1 Fig — Peripheral blood was collected and the enzymes were analyzed in the 1st, 7th and 14th days of treatment. The values are expressed as mean ± s.d. Significance is indicated by: *p<0.05, **p<0.01 and ***p<0.001 compared to control; and #p<0.05, ##p<0.01 and ###p<0.001 compared to untreated normal mice. (TIF) [file pone.0118702.s001.tif]

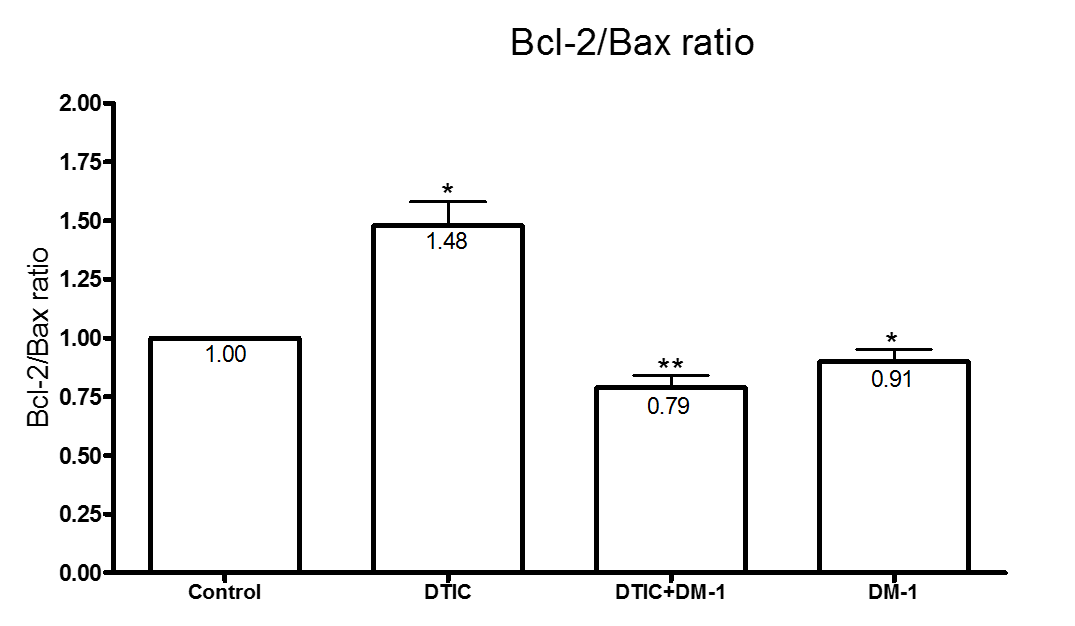

Supplement: S2 Fig — DTIC, DTIC+DM-1 and DM-1 tumor samples were compared to control group. The values are expressed as mean ± s.d. Significance is indicated by: *p<0.05 and **p<0.01 compared to control group. (TIF) [file pone.0118702.s002.tif]
