# Supplementary material for: Curcumin Analog DM-1 in Monotherapy or Combinatory Treatment with Dacarbazine as a Strategy to Inhibit In Vivo Melanoma Progression
Source: PLoS One. 2015 Mar 5;10(3):e0118702. doi: 10.1371/journal.pone.0118702 (PMC4350837; doi:10.1371/journal.pone.0118702)

**Supporting Information**

**Curcumin analog DM-1 in monotherapy or combinatory treatment with dacarbazine as a strategy to inhibit *in vivo* melanoma progression**

Fernanda Faião-Flores, José Agustín Quincoces Suarez, Andréa Costa Fruet, Silvya Stuchi Maria-Engler, Paulo Celso Pardi, Durvanei Augusto Maria


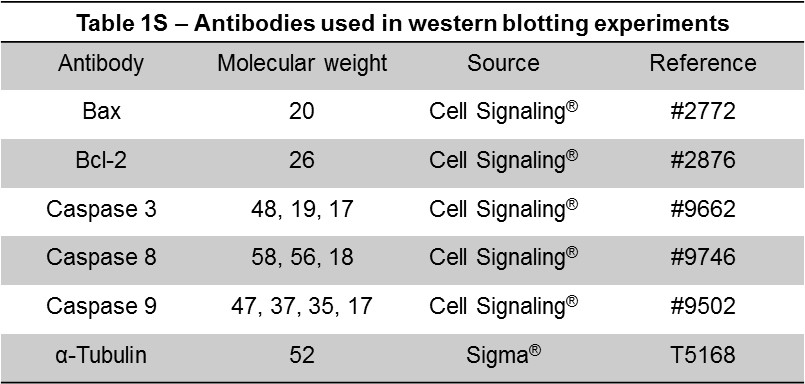

Supplement: S1 Table — (DOC) [file pone.0118702.s003.doc]
